# Bidentatide, a Novel Plant Peptide Derived from *Achyranthes bidentata* Blume: Isolation, Characterization, and Neuroprotection through Inhibition of NR2B-Containing NMDA Receptors

**DOI:** 10.3390/ijms22157977

**Published:** 2021-07-26

**Authors:** Fei Ding, Yunpeng Bai, Qiong Cheng, Shu Yu, Mengchun Cheng, Yulin Wu, Xiaozhe Zhang, Xinmiao Liang, Xiaosong Gu

**Affiliations:** 1 Key Laboratory of Neuroregeneration of Jiangsu and Ministry of Education, Co-Innovation Center of Neuroregeneration, Nantong University, 19 Qixiu Road, Nantong 226001, China; dingfei@ntu.edu.cn (F.D.); cq1981@ntu.edu.cn (Q.C.); yushu@ntu.edu.cn (S.Y.); 2 CAS Key Laboratory of Separation Sciences of Analytical Chemistry, Dalian Institute of Chemical Physics, Chinese Academy of Sciences, Zhongshan Road 457, Dalian 116023, China; bypnenu@126.com (Y.B.); chengmc@dicp.ac.cn (M.C.); wuyulin0525@163.com (Y.W.); 3 School of Pharmacy, Henan University of Chinese Medicine, East Jinshui Road 156, Zhengzhou 450046, China; 4 Partner Group of Max Planck Society, Dalian 116023, China

**Keywords:** enzyme inhibitor, peptide, NMDA receptor, neuroprotection

## Abstract

Increasing attention is being focused on the use of polypeptide-based N-methyl-d-aspartate (NMDA) receptor antagonists for the treatment of nervous system disorders. In our study on *Achyranthes bidentata* Blume, we identified an NMDA receptor subtype 2B (NR2B) antagonist that exerts distinct neuroprotective actions. This antagonist is a 33 amino acid peptide, named bidentatide, which contains three disulfide bridges that form a cysteine knot motif. We determined the neuroactive potential of bidentatide by evaluating its in vitro effects against NMDA-mediated excitotoxicity. The results showed that pretreating primary cultured hippocampal neurons with bidentatide prevented NMDA-induced cell death and apoptosis via multiple mechanisms that involved intracellular Ca^2+^ inhibition, NMDA current inhibition, and apoptosis-related protein expression regulation. These mechanisms were all dependent on bidentatide-induced inhibitory regulation of NR2B-containing NMDA receptors; thus, bidentatide may contribute to the development of neuroprotective agents that would likely possess the high selectivity and safety profiles inherent in peptide drugs.

## 1. Introduction

N-methyl-D-aspartic acid (NMDA) receptors, well known as one class of glutamate-gated ion channels, are critical for regulation of brain function and signal transmission [1]. They are obligatory heterotetramers composed of 2 glycine-binding GluN1 subunits as an essential part and 2 glutamate-binding GluN2 subunits as a regulatory part. As with other ionotropic glutamate receptor subunits, NMDA receptor subunits have a domain-layered architecture, including the amino-terminal domain (ATD), the ligand- or agonist-binding domain (LBD), the transmembrane domain (TMD), and the carboxy-terminal domain (CTD) [2,3,4,5,6]. GluN2A/2B-containing NMDA receptors are the major functional isoforms specific to neurons, and show developmental and regional differences in expression levels [7,8]. GluN2A (NR2A)-containing NMDA receptors are mainly located in synapses in the mature brain and preferentially mediate the cell survival pathway, while GluN2B (NR2B)-containing NMDA receptors reside mainly at extra-synaptic sites and are involved in cell death pathway [9]. Under pathological conditions, overstimulation of NR2B-containing NMDA receptors results in excessive calcium influx and excitotoxicity [10], which may be associated with various neurological diseases and disorders, such as Alzheimer’s disease, stroke, and ischemic damage [6,11,12,13]. Accordingly, NMDA receptors are important targets for drug intervention in nervous system disorders, leading to the development of highly selective antagonists and agonists with differential regulatory functions and fewer side effects.

A number of small molecules have been developed as NMDA receptor modulators. Among them, dizocilpinemaleate (MK-801) is an open channel blocker. It shows neuroprotective actions in animal models of stroke, but it also induces psychotic behavior and neuronal degeneration owing to its high affinity for and long dwell time on NMDA receptors [14]. In contrast, memantine is well tolerated in the clinical as a result of its weaker binding to the ion channel [6,15]. The GNE series of NR2A-selective positive allosteric modulators can be used to indirectly increase the function of NMDA receptors without leading to potentially toxic over activation, which may be therapeutically valuable for neurological disorders involving NMDA receptor hypofunction [16,17]. In contrast, 4-(2-(4-benzylpiperidin-1-yl)-1-hydroxypropyl) phenol (ifenprodil) and its derivatives are NR2B-selective negative allosteric modulators [12], which interact with the ATD of the NR2B subunit through hydrophobic interactions and hydrogen bonds, providing protection in some neurodegenerative diseases [18,19]. In particular, ifenprodil and R-(R,S)-α-(4-hydroxyphenyl)-β-methyl-4-(phenylmethyl)-1-piperidine propranol (Ro5-6981) demonstrate high selectivity in that neither exerts an apparent effect on the ATD of other receptors, such as α-amino-3-hydroxy-5-methyl-4-isoxazolepropionic acid (AMPA) or kainate receptors [19,20,21]. Despite these extensive studies on small molecular neuroprotectants, the search for more numerous and more effective antagonists and agonists of NMDA receptors remains highly necessary.

Recently, a growing interest has emerged towards natural peptide-based drugs used as NMDA receptor modulators, which have superior pharmacological properties over small molecule drugs due to the diversity of their structures and conformations [22,23,24]. To date, different types of polypeptides, including Γ-ctenitoxin-Pn1a (PnTx4-5-5), poly-arginine peptides, and cone snail venom peptides, have been isolated from lower animals and found to interact with NMDA receptors.

PnTx4-5-5, an exciting neurotoxin derived from *Phoneutria nigriventer* spider venom, is a 47 amino acid peptide with 5 pairs of disulfide bonds, which can selectively block NMDA receptors without affecting AMPA, kainite, or γ-aminobutyric acid (GABA) receptors. Evidence shows that PnTx4-5-5 reduces glutamate or amyloid-β induced neuronal cell death [25,26]. Poly-arginine and arginine-rich peptides inhibit calcium influx by decreasing the expression of NR2B-containing NMDA receptors in cultured cortical neurons, displaying neuroprotective actions in an in vitro model of excitotoxicity and in an in vivo model of stroke [27,28]. Cone snail venom peptides are usually classified into disulfide-poor conopeptides and disulfide-rich conotoxins, both having respective specificity for different channels. The disulfide-poor conopeptides (including conantokin-G/T/R/L) are NR2B-selective NMDA receptor antagonists, whereas the disulfide-rich conotoxins are agents that target nicotinic acetylcholine receptors and voltage-gated Na^+^, Ca^2+^, or K^+^ channels [29,30,31,32]. Compared to small-molecule drugs, polypeptide-based drugs represent a new choice for the treatment of NMDA receptor-related diseases with better sensitivity and selectivity.

A series of our previous studies have shown that *Achyranthes bidentata* polypeptides (ABPP), a peptide-rich mixture derived from *Achyranthes bidentata* Blume (a Chinese medicinal herb), can protect cultured hippocampal neurons against NMDA-induced cell apoptosis, exerting bi-directional regulatory effects through inhibition of NR2B-containing NMDA receptors and enhancement of NR2A-containing NMDA receptors [33,34]. ABPP is also able to protect neurons from serum or glucose deprivation in vitro and in vivo [35,36]. Besides the central nervous system, the peripheral nervous system is also affected by ABPP, which promotes nerve regeneration after sciatic nerve injury [37,38,39]. ABPPk was also isolated from *Achyranthes bidentata* Blume by our research team. It possesses stronger neural activity than ABPP [39,40,41] and has a potential anti-Parkinson activity [42]. Unfortunately, ABPP and ABPPk are both peptide mixtures, meaning we were unable to figure out which peptide is the crucial active compound. Would further refined separation yield a pure single peptide as the major active ingredient responsible for all neural activity of *Achyranthes bidentata* Blume? This question may certainly be an interesting issue worth exploring.

It was this question that inspired us to undertake this study, in which a single peptide was isolated and purified from *Achyranthes bidentata* Blume using reverse-phase high-performance liquid chromatography (RP-HPLC) techniques. This new compound, named bidentatide (*Achyranthes bidentata* peptide), was subjected to mass spectrometry (MS) and Edman degradation for composition analysis, and contains 33 amino acid residues with 3 disulfide bonds. So far, bidentatide is the sole active component that we have found and identified from this plant. The neural activity assessments showed that bidentatide prevented primary cultured hippocampal neurons from NMDA-induced cell damage through inhibiting NR2B-containing NMDA receptors, suggesting its potential pharmaceutical applications in the treatment of nervous system disorders.

## 2. Results

### 2.1. Isolation and Identification of Bidentatide from Achyranthes bidentata Blume

**Figure 1:**
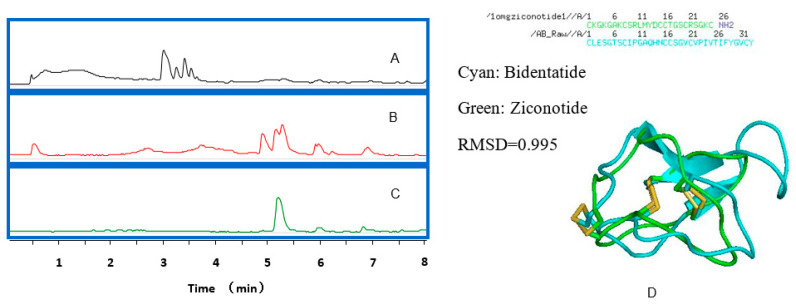
The LC-MS chromatograms of: (**A**) the extract of *Achyranthes bidentata* Blum using 50% methanol; (**B**) the enriched peptides from *Achyranthes bidentata* Blum; (**C**) bidentatide after purification; (**D**) the linear sequence of bidentatide and its homologue, ziconotide (up), as well as their simulated 3D structures.

Bidentatide is a single peptide isolated from *Achyranthes bidentata* Blume (Figure 1). Its amino acid composition was assessed using a combination of Edman degradation and tandem mass spectrometry (MS/MS) (Figure S3). The peptide sequence was deduced from analysis of the MS fragmentation patterns. After dithiothreitol (DTT) reduction and iodoacetamide (IAM) alkylation, electrospray ionization (ESI)–MS/MS data indicated that the molecular weight difference between the IAM derivative of bidentatide and bidentatide itself was 348.16 Da, which resulted from IAM alkylation of 6 cysteines in terms of a molecular weight gain of 57.0215 Da per cysteine residue.

Furthermore, a stepwise approach, designed for analyzing the disulfide linkage pattern in a peptide containing multiple closely spaced cysteine residues [43], was used to identify the disulfide connectivity in bidentatide with the aid of PEAKS search software. Following partial reduction with a low concentration of tris-(2-carboxyethyl)-phosphine (TCEP) and alkylation with N-ethylmaleimide (NEM), the disulfide bond at a relatively more exposed location on the peptide chain was selectively reduced by TCEP and the resultant sulfhydryl groups were tagged (blocked) by NEM, thus determining the location of a disulfide bond (Table 1). A successive application of these procedures helped to identify 3 pairs of disulfide bonds using PEAKS searching, which existed between Cys1-Cys17, Cys8-Cys21, and Cys16-Cys32 residues, respectively (Figures S2 and S3). At this point, we deduced that bidentatide was a 33-mer peptide with 3 disulfide bridges (Figure S2). The cysteine bonding pattern belongs to the inhibitor cystine knot (ICK) family, including Cys (I-IV), Cys (II-V), and Cys (III-VI), which has been found in plant peptides of diverse origins [44].

### 2.2. Effects of Bidentatide on NMDA-Induced Neuronal Death

After cultured hippocampal neurons were exposed to 100 μM NMDA for 30 min, the cell viability was significantly decreased. Pretreatment with bidentatide (25–200 nM), however, attenuated the NMDA-induced decrease of cell viability in a concentration-dependent manner (Figure S4). MK-801 (10 μM), serving as a positive control, also blocked NMDA-induced excitotoxicity and produced a similar effect to that of 100 or 200 nM bidentatide.

### 2.3. Effects of Bidentatide on NMDA-Induced Calcium Influx

**Figure 2:**
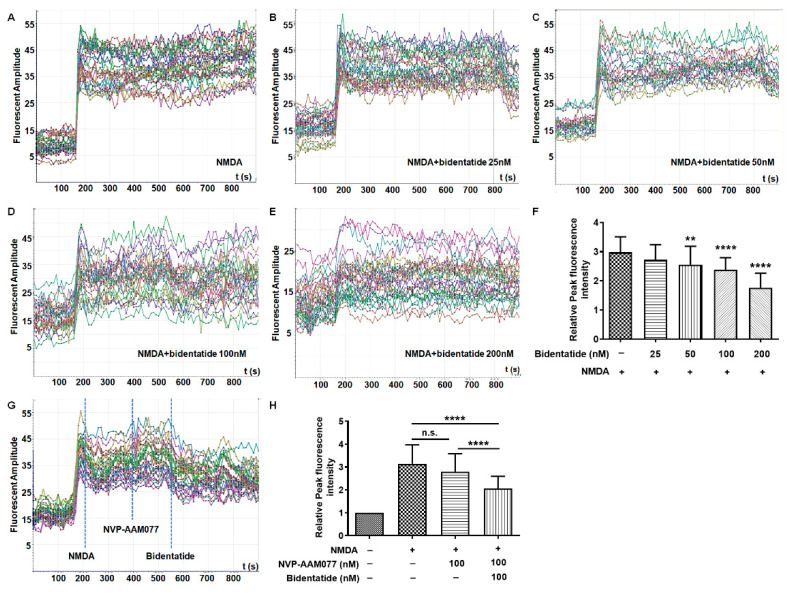
Bidentatide effects on NMDA-induced Ca^2+^ overload in cultured hippocampal neurons: (**A**–**E**) dynamic changes of intracellular Ca^2+^ levels in primary cultured hippocampal neurons upon pretreatment with different concentrations of bidentatide and exposure to NMDA (100 μM); (**F**) bar graph showing the relative fluorescence intensity of NMDA-induced Ca^2+^ in primary cultured hippocampal neurons; **** *p* < 0.0001 and ** *p*< 0.01 versus exposure to NMDA alone; (**G**) dynamic changes of intracellular Ca^2+^ levels after the addition of 100 μM NMDA, 100 nM NVP-AMM077, and 100 nM bidentatide; the dotted blue line represents the peak of fluorescence intensity; (**H**) bar graph showing the relative fluorescence intensity of NMDA-induced Ca^2+^ in primary cultured hippocampal neurons; **** *p*< 0.0001; n.s. (no significance).

In order to verify the selective inhibition of NMDA receptors by bidentatide, Fluo-4/AM was used to detect NMDA-induced calcium influx in cultured hippocampal neurons after different cell treatments. Combination treatment with NMDA and bidentatide retarded NMDA-induced [Ca^2+^]_i_ elevation in a concentration-dependent manner (Figure 2A–F). Treatment with 100 nM (R)-[(S)-1-(4-bromo-phenyl)-ethyl amino]-(2,3-dioxo-1,2,3,4-tetrahydroquinoxalin-5-yl)-methyl]-phosphonic acid (NVP-AAM077, a NR2A-preferring antagonist) slightly alleviated NMDA-induced [Ca^2+^]_i_ elevation (*p* > 0.05), while treatment with a further 100 nM bidentatide significantly reduced NMDA-induced [Ca^2+^]_i_ influx (*p* < 0.0001, Figure 2G,H), suggesting that the effects of bidentatide were closely related to the inhibition of 2B-containingNMDA receptors.

### 2.4. Effects of Bidentatide on NMDA-Evoked Currents

**Figure 3:**
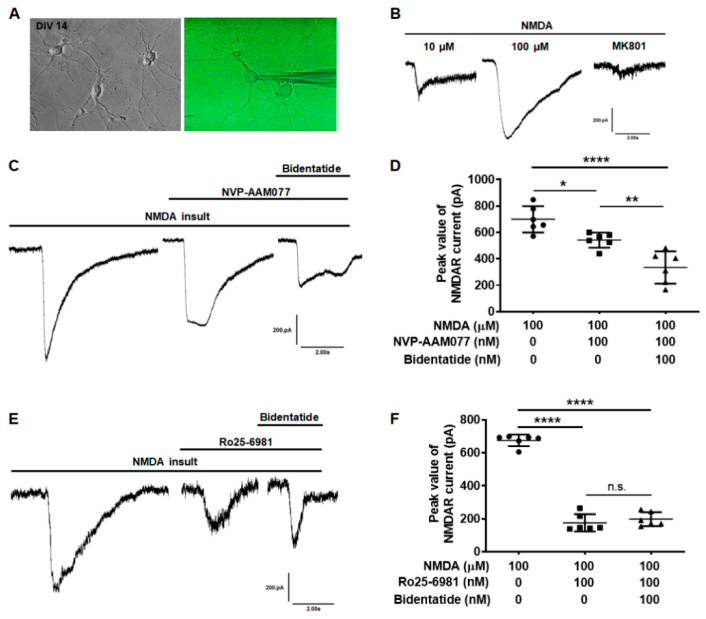
Bidentatide effects on NMDA evoked currents in primary cultured hippocampal neurons: (**A**) the cell morphology of primary cultured hippocampal neurons (DIV14), as observed under phase contrast microscope (left) or as clamped with glass electrode connected to patch clamp amplifier (right); (**B**) effects of MK801 (10 μM) on NMDA-evoked current; (**C**) effects of bidentatide (100 nM) on NMDA-evoked current with NR2A blocked by NVP-AAM077 (100 nM); (**D**) bar graph showing bidentatide (100 nM) effects on the peak value of NMDA-evoked current with NR2A blocked by NVP-AAM077 (100 nM); **** *p* < 0.0001, ** *p* < 0.01, * *p* < 0.05; (**E**) effects of bidentatide (100 nM) on NMDA receptor current with NR2B blocked by Ro25-6981 (100 nM); (**F**) bar graph showing bidentatide (100 nM) effects on the peak value of NMDA-evoked current with NR2A blocked by Ro25-6981 (100 nM); **** *p* < 0.0001; n.s. means no significance.

To further verify the regulatory effect of bidentatide on NMDA receptors, whole-cell patch clamp was used to record the NMDA-evoked currents in cultured hippocampal neurons (Figure 3A). The inward current was rapidly evoked by 10 μM NMDA in the Mg^2+^-free solution and the current was significantly increased with the increase of NMDA concentration (in the range studied). Pretreatment with MK801 (10 μM) significantly blocked NMDA-evoked currents owing to its binding to NMDA receptors (Figure 3B). The inward current in cultured hippocampal neurons was rapidly evoked by NMDA (100 μM) in the absence of Mg^2+^. Pretreatment with 100 nM NVP-AAM077 alone reduced the NMDA-evoked current (*p* < 0.05), which was only partially but not completely decreased. Combined pretreatment with 100 nM bidentatide and 100 nM NVP-AAM077 yielded a greater decrease in NMDA-evoked current compared to pretreatment with 100 nM NVP-AAM077 alone (*p* < 0.01, Figure 3C,D). On the other hand, pretreatment with 100 nM Ro25-6981 (a NR2B antagonist) alone significantly reduced the NMDA-evoked current (*p* < 0.0001). Combined pretreatment with 100 nM bidentatide and 100 nM Ro25-6981 did not lead to a greater decrease in NMDA-evoked current compared to pretreatment with 100 nM Ro25-6981 alone (Figure 3E,F), suggesting that the effects of bidentatide were likely through inhibition of NR2B.

### 2.5. Effects of Bidentatide on NMDA-Induced Neuronal Apoptosis

**Figure 4:**
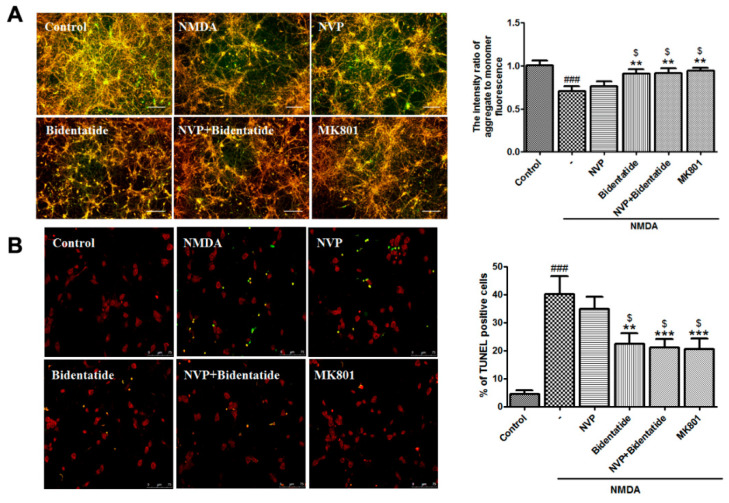
Involvement of NR2B-containing NMDA receptors in anti-apoptosis effects of bidentatide: (**A**) representative fluorescence micrographs of JC-1 staining for (aggregate in red, monomer in green, and the merge in yellow) and bar graph of the fluorescence intensity ratio of aggregate-to-monomer in primary cultured hippocampal neurons upon no treatment, exposure to NMDA alone, pretreatment with NVP-AAM077 (100 nM) for 30 min and then exposure to NMDA, pretreatment with bidentatide (100 nM) for 30 min and then exposure to NMDA, pretreatment with NVP-AAM077 (100 nM) and bidentatide (100 nM) for 30 min and then exposure to NMDA, and pretreatment with 10 μM MK801 for 30 min and then exposure to NMDA; scale bar, 100 μm; (**B**) representative fluorescence micrographs of TUNEL staining (PI staining in red, TUNEL-positive staining in green, the merged area in yellow) and bar graph of the percentage of TUNEL-positive cells in primary cultured hippocampal neurons upon different treatments as mentioned above. Yellow indicates merged PI staining (red) and TUNEL-positive staining (green); scale bar, 75 μm; ^###^ *p* < 0.001 verses control; ** *p* < 0.01, and *** *p* < 0.001 verses NMDA alone; ^$^ *p* < 0.05 versus pretreatment with NVP-AAM077 plus NMDA.

Excitotoxic neuronal injury is mediated by a cascade of apoptosis [45]. The loss of mitochondrial membrane potential (MMP), as an early apoptotic event, represents an available index of cell apoptosis [46]. To examine the effect of bidentatide on NMDA-induced MMP loss, 100 µM NMDA was applied to induce MMP depolarization of cultured hippocampal neurons. Pretreatment with 100 nM NVP-AAM077 alone slightly attenuated NMDA-induced MMP decrease without significant differences. In contrast, pretreatment with bidentatide (100 nM) or combined pretreatment with 100 nM NVP-AAM077 and 100 nM bidentatide significantly ameliorated NMDA-induced MMP decrease, exhibiting the stronger increase of MMP than pretreatment with 100 nM NVP-AAM077 alone (*p* < 0.05, Figure 4A). At the later steps of cell apoptosis, the morphological changes of cells, e.g., nuclear DNA breakage and nuclear pyknosis, can be observed [47]. TdT-mediated dUTP nick-end labeling (TUNEL) assay indicated that after NMDA stimulation, a number of cultured hippocampal neurons displayed nuclear shrinkage, and the percentage of TUNEL-positive cells in all cells was elevated. Pretreatment with 100 nM NVP-AAM077 alone exerted a blocking effect on TUNEL-positive cell increase but failed to produce a significant outcome. In contrast, the number of TUNEL-positive cells resulting from pretreatment with bidentatide or combined pretreatment with NVP-AAM077 and bidentatide was significantly lower than that resulting from pretreatment with NVP-AAM077 alone (*p* < 0.05, Figure 4B). Moreover, the anti-apoptosis effect of bidentatide was close to that of 10 µM MK801 (Figure 4A,B). In summary, the data indicated that inhibition of neuronal apoptosis by bidentatide was mainly linked to its interactions with NR2B rather than the NR2A subunit.

**Figure 5:**
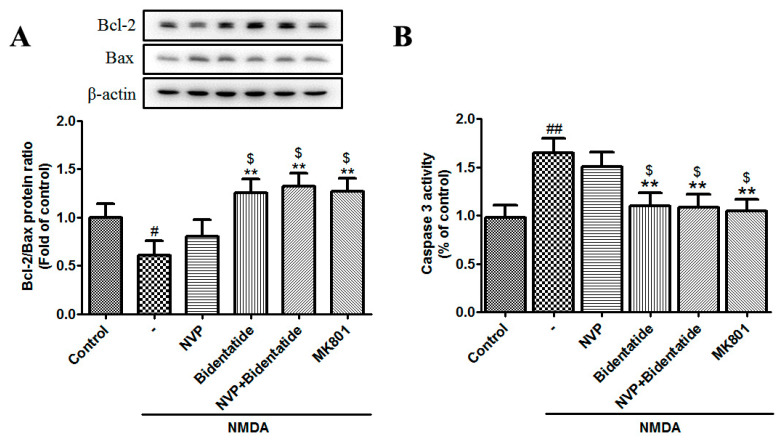
Involvement of NR2B-containing NMDA receptors in bidentatide regulation of apoptosis-related protein expression: (**A**) representative Western blot images and the related bar graph showing the Bcl-2/Bax expression ratio in primary cultured hippocampal neurons upon different treatments, as mentioned in Figure 4A; (**B**) bar graph showing the activity of caspase 3 in primary cultured hippocampal neurons upon different treatments, as mentioned in Figure 4A; ^#^ *p* < 0.05 and ^##^ *p* < 0.01 verses control; ** *p* < 0.01 verses NMDA alone; ^$^ *p* < 0.05 versus pretreatment with NVP-AAM077 plus NMDA.

The mitochondrial-mediated apoptotic cascade is mainly regulated by caspase and Bcl-2 family members [48]. The Bcl-2 family includes anti-apoptotic proteins (e.g., Bcl-2 and Bcl-xL) and pro-apoptotic proteins (e.g., Bax, Bad, Bak, Bik, and Bcl-xs) [49]. The balance between Bcl-2 and Bax and the activation of caspase 3 (an apoptotic executioner) play key roles in apoptotic progress [50]. Our data indicated that NMDA stimulation reduced the Bcl-2/Bax ratio and enhanced caspase 3 activity in cultured hippocampal neurons, while pretreatment with bidentatide or combined pretreatment with NVP-AAM077 and bidentatide more strongly enhanced the Bcl-2/Bax ratio and reduced caspase 3 activity as compared with pretreatment with NVP-AAM077 alone (*p* < 0.05, Figure 5A,B). Taken together, these data provided further evidence for the involvement of the NR2B subunit in the neuroprotection of bidentatide against neuronal excitotoxicity.

## 3. Discussion

Glutamate is the most important excitatory transmitter in the mammalian CNS [51]. The glutamate receptors are divided into 2 major families, namely ionotropic glutamate receptors (coupled to the ionic channel) and metabotropic glutamate receptors (bound to G-protein); the former family includes NMDA, AMPA, and kainate receptors [52]. Compared to other ionotropic receptors, NMDA receptors have received more attention in that they have complex subunit constitution and unique biological properties, thereby playing a central role in the CNS development and function, especially in synaptic plasticity and excitatory toxicity. NMDA receptors are involved in glutamate-mediated neuronal survival and death pathways [8,53,54]. Interestingly, activation of either synaptic or extra-synaptic NR2B-containing NMDA receptors results in excitotoxicity and neuronal apoptosis, but activation of either synaptic or extra-synaptic NR2A-containing NMDA receptors promotes neuronal survival and produces neuroprotection against both NMDA and non-NMDA-receptor-mediated neuronal damage [9]. These phenomena are more or less responsible for the complexity of using the NMDA modulator as a drug target for treating the CNS disorders [55,56], and also provide an idea on how to develop specific NMDA-modulator-based therapies.

Our previous studies focused on the search for neuroprotective agents of plant origin. In particular, we reported that ABPP derived from *Achyranthes bidentata* Blume, a Chinese medicinal herb, can confer selective neuroprotection in diverse in vitro and in vivo models through NMDA-receptor-dependent mechanisms [33,34,35,36,37,38,39,40,41,42]; however, the ABPP product is a mixture of polypeptides and may be intermingled with a small amount of non-active or adverse constituents, which are bound to have a diminishing impact on the pharmacological efficacy of bidentatide. Although purification of a single peptide from plant extracts is a very challenging task due to its small proportion in the mixture and interferences of impurities, in this study a single peptide bidentatide was isolated and purified from *Achyranthes bidentata* Blume by means of sequential RP-HPLC separations.

According to amino acid sequencing, bidentatide is a 33 amino acid peptide with 3 disulfide bridges formed by 6 cysteines. This amino acid composition results in the low polarity of the molecule, which is predominantly positively charged, as evidenced by the theoretical pI value of 5.24. This ICK motif has been found in a variety of short peptides (usually <40 residues) derived from plants and animals (e.g., cone snails, scorpions and spiders) [20,57]. These peptides include ionic channel-binding toxins, trypsin and α-amylase inhibitors, antimicrobial peptides, and sweet-taste-suppressing plant peptides, and exhibit distinct biological behaviors [58]. The triple-stranded antiparallel β-sheets occurring in the ICK-motif-sharing peptides are different from each other in the lengths of their loops and turns and the numbers of involved residues. We note that the cysteine arrangement of bidentatide (C-C-CC-C-C) is consistent with that of the conotoxins [59].

NMDA-induced neuronal apoptosis is mediated by overload of intracellular Ca^2+^ due to overstimulation of NMDA receptors and plays an important pathophysiological role in CNS disorders, including trauma, stroke, ischemia, and chronic neurodegenerative diseases [10]. NMDA receptors can be regulated by numerous ligand-recognition sites, including the sites for the agonist L-glutamate, the sites within the pore of ion channels, and the sites for allosteric modulators of receptor function (e.g., polyamines, steroids, metal ions, and protons) [60]. The neuroactivity test data may suggest the inhibitory modulation of NR2B-containing NMDA receptors by bidentatide.

To investigate the possible neuroprotective function of bidentatide, primary cultured hippocampal neurons, as an in vitro model system, were exposed to NMDA stimulation. An NMDA-induced decrease in neuronal survival was ameliorated by pretreatment with bidentatide or MK801 (a common NMDAR antagonist). Since NMDA-induced neuronal damage usually takes the form of cell apoptosis, we further examined the protective action of bidentatide against NMDA-induced apoptosis and observed that NMDA-induced MMP depolarization and apoptotic cell increase were attenuated by pretreatment with bidentatide or MK801.

Since the key step in NMDA-induced neuronal apoptosis is intracellular Ca^2+^overload, Fluo-4 AM imaging was used to monitor the alterations of intracellular Ca^2+^ in cultured hippocampal neurons upon different cell treatments. Treatment with bidentatide or MK-801 significantly retarded the NMDA-induced increase in Ca^2+^ influx, while bidentatide demonstrated a concentration-dependent effect. Interestingly, treatment with NVP-AAM007 (a NR2A preferential antagonist) alone slightly (not significantly) reduced the NMDA-induced increase in [Ca^2+^] influx, followed by treatment with bidentatide, which significantly reduced NMDA-induced increase in Ca^2+^ influx, suggesting that bidentatide inhibited intracellular Ca^2+^ influx through antagonizing NR2B.

By using whole-cell patch clamp recording techniques, we observed that bidentatide itself had no effect on electrophysiological properties of primary cultured hippocampal neurons. When NMDA was used to induce neuron excitatory toxicity, the NMDA-evoked current significantly increased, suggesting that the NMDA receptor channel was in a highly active state. Pretreatment with NVP-AAM007 alone partially reduced the NMDA-evoked current elevation by blocking NR2A subunits, while bidentatide further inhibited the NMDA-evoked current elevation in the presence of NVP-AAM007 through inhibiting the NR2B subunit on the neuron surface. On the other hand, Ro25-6981 (a NR2B antagonist) inhibited the NMDA-evoked current elevation, while bidentatide could not affect the NMDA current change in the presence of Ro25-6981; therefore, whole-cell patch clamp recordings provided further evidence for bidentatide inhibition of the NR2B subunit on the neuron surface.

To better elucidate the possible mechanism underlying the anti-neuronal apoptotic function of bidentatide, we noted that bidentatide enhanced the expression ratio of Bcl-2 (an anti-apoptotic protein) and Bax (a pro-apoptotic protein) and inhibited the activation of caspase 3 (an apoptotic executor). These observations indicated that bidentatide played a positive role in antagonizing excitotoxicity-mediated neuronal apoptosis. Moreover, intervention with NVP-AAM077 did not exert significant anti-apoptotic effects on cultured hippocampal neurons exposed to NMDA. Combined pretreatment with bidentatide and NVP-AAM077 effectively blocked neuronal apoptosis, suggesting that the NR2B subunit was involved in the anti-neuronal apoptotic function of bidentatide.

Perhaps, it may be questioned why pretreatment with NVP-AAM077 alone could slightly inhibit NMDA-induced excitotoxicity, because neuroprotection usually benefits from activation rather than antagonism of the NR2A subunit, while NVP-AAM077 is an NR2A antagonist. In fact, recent evidence challenges the early assumption that NVP-AAM077 displays very strong selectivity for NR2A, and indicates that the subunit selectivity of NVP-AAM077 is only 5–10-fold higher for NR2A than for NR2B [61,62]; therefore, although the well-documented effect of NVP-AAM077 is preferential blockade for NR2A, it is still able to inhibit NR2B to a certain extent, having a moderate neuroprotective role. Although these pharmacological results demonstrate the neuroprotection of the bidentatide, the role of bidentatide may demand continuous analysis in physiological environments.

## 4. Materials and Methods

Described here are the materials used in the following 3 subsections. Bidentatide crude extract was prepared from *Achyranthes bidentata* Blume as we previously described [33] and stored at −80 °C until use. Acetonitrile, formic acid, methanol, and ammonium acetate (all HPLC-grade) were purchased from Sigma (St. Louis, MO, USA). Chloroform, n-butanol, ammonium, and hydrochloric acid were purchased from Kemiou (Tianjin, China). Reduced and derivative reagents, including DTT, IAM, TCEP, and NEM, were obtained from Aladin (Shanghai, China). Urea and tris-(hydroxymethyl)-aminomethane (Tris) buffer were supplied by Solarbio (Beijing, China). Ultra-pure water was prepared using a Milli Q system (Bedford, MA, USA) and deuterated water was obtained from HeownsBiochem LLC (Tianjin, China) with a purity of 99.9%.

### 4.1. Isolation and Purification of Bidentatide

Roots of A. bidentata (2 kg) were purchased from a local Chinese medicine grocer and identified by the pharmacist. After being cold-soaked overnight at 4 °C, the roots were heated for 2 h at 80 °C. The decoction was saturated with ammonium sulfate to 50% saturation and fully stirred in the reactor at 4 °C for 12 h. The precipitate was discarded by centrifugation at 4 °C and 15,000 rpm for 30 min. The supernatant was collected and saturated with ammonium sulfate to 80% saturation. After being fully stirred in a reactor at 4 °C for 12 h, the supernatant was removed by centrifugation at 4 °C for 30 min at 15,000 rpm. The left precipitate was dissolved in sterilized water to undergo dialysis in 1000 Dalton molecular weight cut-off (MWCO) tubings with ultrapure water at 4 °C for 1 week. The extractions were combined and concentrated.

In total, 2 g crude peptide extract was used to purify bidentatide. Sevage reagent was used with chloroform/n-butanol at a volume ratio of 5:, while the volume ratio for Sevage reagent/aqueous extract solution was 1:3. The water layer was extracted twice and the intermediate solid layer was combined. A preparative C18 column (innoval ods-p, 5 μm, 100 Å, 21.2 × 250 mm, Agela technologies) was used to separate peptides. The mobile phase was 70% methanol water solution (containing 0.5% formic acid). The concentrated peptide was purified on an analytical C18 column (cosmosil, 5 μm, 4.6 × 250 mm) with a binary mobile phase. The mobile phase was an aqueous solution containing 0.5% formic acid, while the mobile phase B was acetonitrile. The elution gradient was 0–8 min, 30–70% B, 8.1–9 min, 100% B, 9.1–10 min, 30% B, the flow rate was 1 ml / min, the retention time was about 6.5 min, and the samples were collected and concentrated according to the retention time. The peptide fraction was then purified on a YMC pack diol NP column (5 μm, 10 × 250 μm). The mobile phase was 80:20 acetonitrile/200 mM ammonium acetate solution, while the flow rate was 4ml/min. The peptide was collected and concentrated before desalting. The purity of bidentatide was analyzed using Q-TOF-MS (Angilent 6520).

### 4.2. Amino Acid Sequencing of Bidentatide

A combination of tandem MS and Edman degradation were used to deduce the composition information for bidentatide. Since cysteine is the only amino acid without any reference for comparison in Edman degradation, automated Edman degradation was performed to identify the amino acid sequence of bidentatide using a PPSQ-51A protein sequencer (Shimadzu, Kyoto, Japan). Thermo Orbitrap Elite was used to analyze the primary amino acid sequence of the purified peptide (Table S1). Figure S1A shows the secondary mass spectrum of the active peptide of Achyranthes bidentata (Table S2). Two peptide sequences PIVTIFYGV and PGAQHN can be identified. The classical derivatization reagent DTT/iodoacetamide (IAM) [63] was used for the reduction and derivatization of cysteine in the disulfide bond of Achyranthes bidentata active peptide. The mass of each disulfide bond of Achyranthes bidentata active peptide increased by 2 × 58.0293 after full derivatization. Figure S1B shows the HCD secondary mass spectrum of the IAM derivative of Achyranthes bidentata active peptide (Table S3), showing the peptide sequence CVPIVTIFYGVCY; the CY sequence of the C-terminal was determined by the mass spectrum data from acid hydrolysis carried out for 5 min, as shown in Figure S1C and Table S4. Based on Edman degradation and mass spectrometry analysis, the primary amino acid sequence of Achyranthes bidentata active peptide was CLESGTSCIPGAQHNCCSGVCVPIVTIFYGVCY, named as bidentatide (containing three disulfide bonds, Figure S2).

### 4.3. Disulfide Bond Connection Pattern of Bidentatide

Under acidic conditions using 0.5% formic acid, bidentatide was mixed with 50 μL of 25 mM TCEP and placed at room temperature for 1 h. The mixture was then added to a 4-fold volume of NEM and placed in a 65 °C water bath for an additional 0.5 h to accomplish stepwise reduction of disulfide bonds and alkylation of free sulfhydryl groups. Desalting was performed in an Amicon Ultra-4 centrifugal filter device with a MWCO of 3000 Da (Merck Millipore Ltd. Darmstadt, Germany). In order to diminish the unreduced disulfide bonds, an excess of TCEP was again added for reaction in a 65 °C water bath before ultra-performance liquid chromatography (UPLC)-Orbitrap–MS analysis on a Waters Acquity Ultra-Performance system (Waters) coupled to a LTQ-Orbitrap Elite spectrometer via an ESI source (Thermo Scientific). Chromatographic separation was performed on a Poroshell 120 EC-C_18_ column (50 mm × 2.1 mm, 2.7 µm, Agilent, Palo Alto, CA, USA) at 45 °C. The other parameters were the same as those used for acid hydrolysis.

The raw data for bidentatide and its derivatives were uploaded to PEAKS Studio 7 software (Bioinformatic Solutions, Waterloo, Canada) for structural characterization. Regarding the error tolerance control, the mass tolerance levels for parent ions and product ions were set at 10 ppm and 0.1 Da, respectively. The filters with quality values > 0.3 were kept for sequence analysis, while the processing procedures, including the peak centroiding, charge deconvolution, and deisotoping procedures, were carried out to ensure data refinement. No enzyme was specified for cleavage. Variable post-translational modification of NEM (Δ mass of +125.0477) on a cysteine residue was selected in the database search and data were retrieved in FASTA format (GenBank, ftp://ncbi.nlm.nih.gov/genbank/genomes, accessed on 20 April 2021). The cut-off of the false discovery rate for peptide identification in the PEAKS search was set at <1%. The structures were displayed and analyzed using homologous modeling and the PyMol program (www.pymol.org, accessed on 20 April 2021).

### 4.4. Cell Culture

Primary cultured hippocampal neurons were obtained from embryonic day 18 (E18) Sprague–Dawley (SD) rats as previously described [64]^.^ After the animals were sacrificed by cervical dislocation under anesthesia, their brains were quickly removed and the hippocampi were harvested on a cold stage. The procured hippocampal tissue was mechanically and enzymatically digested into a single-cell suspension. The cells were re-suspended in DMEM supplemented with 10% fetal bovine serum (FBS) and plated onto poly-lysine-coated plates for incubation in humidified atmosphere of 95% air and 5% CO_2_ at 37 °C. Four hours later, the medium was replaced by serum-free neurobasal medium containing 2% B27 supplement, 0.25% GlutaMAX, 100 U/mL penicillin, and 100 mg/mL streptomycin (all from Gibco, Grand Island, NY), then the cells were incubated for 12–14 days. Half of the culture medium was changed twice a week.

### 4.5. Cell Treatment

Primary cultured hippocampal neurons were washed twice using Mg^2+^-free Hank’s solution, then moved to Mg^2+^-free Hank’s solution supplemented with 100 μM NMDA for 30 min. NMDA stimulation was terminated by washing the cells twice and allowing the cells access to normal culture medium for 24 h incubation. Bidentatide, NVP-AAM077, Ro25-6981, or MK-801 was respectively added to the culture medium 30 min prior to NMDA and kept throughout the excitotoxic injury. Bidentatide was dissolved in ultra-pure water at a concentration of 100 μM to give a stock solution, which was diluted to produce the working solutions at indicated concentrations by referring to our previous data [33,34,41,42]. MK-801(non-specific NMDA receptor inhibitor) was supplied by Sigma, whereas both NVP-AAM077 (a specific NR2A inhibitor) and Ro25-6981 (a specific NR2B inhibitor) were supplied by MedChemExpress, LLC (Shanghai, China). The stock solutions of NVP-AAM 077 and MK-801 (both at 100 mM) were prepared with ultra-pure water, whereas Ro25-6981 was first dissolved in DMSO and then diluted in ultra-pure water to give a stock solution (200 mM).

### 4.6. Cell Viability Analysis

The cultured hippocampal neurons were plated in 96-well cell culture clusters. The cell viability was assessed by CCK-8 method according to the manufacturer’s protocol (Dojindo Molecular Technology, MD, USA). Briefly, tetrazolium salt-8 (WST-8) solution was added to cell culture (10 μL/each well), followed by incubation at 37 °C for 2 h. The absorbance (optical density, OD, USA) was measured by spectrophotometry at 450 nm with an ELx-800 microplate reader (Bio-TekInc., Winooski, VT, USA). The cell viability was expressed as a percentage of control value.

### 4.7. MMP Assessment

MMP assessment was performed using the JC-1 probe as described previously [40]. Briefly, primary cultured hippocampal neurons at DIV12 were incubated with 2 µM fluorescent probe JC-1 (Sigma) at 37 °C for 15 min, followed by washing 3 times with serum-free DMEM to remove excessive probes. The fluorescent photograph was captured under an inverted microscope (IX-71, Olympus, Tokyo, Japan). Quantitative analysis were performed using the Synergy H1 Hybrid Microplate Reader (BioTek, Winooski, VT). For JC-1 monomer or aggregate, excitation and emission of the fluorescence detector were set to 490 and 530 nm or 525 and 590 nm, respectively.

### 4.8. TUNEL Assay

TUNEL assay was performed using a TUNEL kit (Promega, Madison, WI, USA), as per the kit instructions. In brief, primary cultured hippocampal neurons were fixed with 4% formaldehyde for 20 min, permeabilized in 0.2% Triton X-100, equilibrated in equilibration buffer for 10 min, and then incubated in a buffer mixture containing recombinant terminal deoxynucleotidyltransferase (rTdT) and nucleotide at 37 °C in the dark for 1 h. The cell nuclei were counterstained with propidium iodide (PI) for 5 min. TUNEL-positive signaling was visualized under a confocal microscope (Leica, Heidelberg, Germany). Data are expressed as the ratio of apoptotic cells to total cells.

### 4.9. Intracellular Calcium Measurement

Scanning laser confocal microscopy (Leica TCS SP2, Wetzlar, Germany) was used to determine cytosolic Ca^2+^ in primary cultured hippocampal neurons at day in vitro 12 (DIV12). The cells were loaded with 5 μM Fluo-4/AM (Beyotime, Shanghai, China) in Hank’s solution at 37 °C in the dark for 45 min, then washed using Hank’s solution gently to remove extracellular Fluo-4/AM dye. Fluorescence was monitored immediately every 10 s over a period of 15 min (excitation at 488 nm and emission at 526 nm). Prior to NMDA stimulation, the dye-loaded cells were scanned for ~2 min to obtain a basal level of fluorescence intensity. After different cell treatments as mentioned above, the fluorescence intensity was monitored and the dynamic change of the intracellular Ca^2+^ concentration ([Ca^2+^]_i_) was recorded. The [Ca^2+^]_i_ value was measured according to the relative fluorescence intensity to the basal level.

### 4.10. Electrophysiology

To record NMDA-evoked currents, whole-cell patch clamp recordings were obtained from primary cultured hippocampal neurons at DIV14. The cells were bathed in a Mg^2+^-free bathing solution (in mM: NaCl 140, KCl 5, CaCl2 2.5, HEPES 10, and D-glucose 10, pH 7.25, osmotic pressure 310 mOsm, all reagents from Sigma), which was supplemented with 1 μM tetrodotoxin (TTX, a sodium channel blocker, sigma) and 50 μM bicuculline. Recording pipettes were filled with an intracellular solution (in mM: K-gluconate 120, NaCl 5, MgCl_2_ 1, EGTA 0.2, MgATP 2, Na_3_GTP 0.1, HEPES 10, and phosphocreatine disodium 10, pH 7.25, osmotic pressure 310 mOsm; all reagents from Sigma). Recordings were performed at room temperature in voltage-clamp mode at a holding potential of −70 mV using an EPC 10 USB patch clamp amplifier and a PatchMaster software (both from HEKA Elektronik, Ludwigshafen, Germany). NMDA (100 μM) and glycine (10 μM) were used to activate NMDA receptors. Series resistance <20 MΩ was monitored for consistency during recordings. The signals were amplified, sampled at 10 kHz, filtered at 3 kHz, and analyzed using PatchMaster software.

### 4.11. Western Blot Analysis

Total proteins were extracted and quantified using routine protocols. The samples were separated using 12% SDS-PAGE gel, then transferred into PVDF membranes. After 5% skim milk blocking, membranes were reacted with primary antibodies against Bax, Bcl-2, or β-actin (1:1000, Abcam, Cambridge, UK) at 4 °C overnight, then with HRP-conjugated antibody (1:5000) at room temperature for 1 h. Immunoreactive bands were visualized with a BeyoECL Plus Kit (Beyotime, Nantong, China). Protein quantification was performed using ImageJ software (NIH Bethesda, MD, http://www.rsb.info.nih.gov/ij, accessed on 20 April 2021).

### 4.12. Caspase 3 Activity Assay

Caspase 3 activity assay was performed on primary cultured hippocampal neurons using the caspase 3/CPP32 colorimetric Assay Kit (BioVision, Milpitas, CA, USA), as per the kit protocols. In brief, the cells were collected and re-suspended in a cell lysis buffer. After centrifugation, the supernatant was collected and quantified using a BCA Protein Assay Kit (Beyotime, Shanghai, China). The sample was added with a reaction buffer containing 10 mM dithiothreitol (DTT) and 4 mM of Ac–Asp–Glu–Val–Asp–chromophore p–nitroanilide (Ac-DEVD-pNA) and incubated at 37 °C for 2 h. The absorbance at 405 nm was measured using the Synergy H1 Hybrid Microplate Reader (BioTek).

### 4.13. Statistical Analysis

Data are expressed as means ± SD. The GraphPad Prism 6.0 software package was used for statistical analysis. For normally distributed variables, one-way ANOVA and Bonferroni post hoc test were used to compare differences between groups. If only 2 groups were compared, an unpaired 2-tailed Student’s t test was applied. Here, *p* < 0.05 was considered statistically significant.

## 5. Conclusions

A single peptide bidentatide was isolated and purified from the extract of *Achyranthes bidentata* Blume. The amino acid sequence and disulfide connection pattern of bidentatide were identified using Edman degradation and MS/MS technology. To evaluate the neuroactive potential of bidentatide, we observed its effects on NMDA-induced excitotoxicity in vitro and found that pretreatment with bidentatide prevented primary cultured hippocampal neurons from NMDA-induced cell death and apoptosis via multiple mechanisms involving intracellular Ca^2+^inhibition, NMDA current modulation, and apoptosis-related protein expression regulation. All of the above mechanisms were dependent on inhibitory regulation of NR2B-containing NMDA receptors by bidentatide. Overall, this study may contribute to developing promising plant-peptide-based neuroprotectants and to promoting the modernization of traditional Chinese medicine. Moreover, a thorough understanding of the relationship between structure and activity in bidentatide would be beneficial to artificial synthesis of peptide(s)-based drugs with neurotrophic and neuroprotective functions.

## Figures and Tables

**Table 1:** TCEP/NEM step-by-step reduction of bidentatide to obtain disulfide bond information.

**No.**	**Peptide**	**−10lgP**	**Mass**	***m*/*z***	**z**	**RT**	**ppm**
1	C(+125.05)LESGTSCIPGAQHNCC(+125.05)SGVCVPIVTIFYGVCY	16.06	3672.5901	1225.2031	3	3.71	−0.7
2	C(+125.05)LESGTSCIPGAQHNC(+125.05)C(+125.05)SGVCVPIVTIFYGVC(+125.05)Y	55.55	3922.6865	1308.5698	3	3.85	0.3

**No.**

**Peptide**

**−10lgP**

**Mass**

***m*/*z***

**z**

**RT**

**ppm**

## Data Availability

The data generated or analyzed during this study are included in this published article (and its Supplementary Information Files).

## Supplementary Materials

The following are available online at https://www.mdpi.com/article/10.3390/ijms22157977/s1.

## Abbreviations

Bidentatide	*Achyranthes bidentate* Blume polypeptide
ATD	amino-terminal domain
DTT	dithiothreitol
ESI	electrospray ionization
Ifenprodil	4-[2-(4-benzylpiperidin-1-yl)-1-hydroxypropyl]phenol
GABA	γ-aminobutyric acid
HCD	High-collision dissociation
IAM	Iodoacetamide
LC-MS	liquid chromatography mass spectrometry;
MK-801	dizocilpine
MMP	mitochondrial membrane potential
MS/MS	tandem mass spectrometry
MPA	α-amino-3-hydroxy-5-methyl-4-isoxazolepropionic acid
NMDA	N-methyl-D-aspartate
NVP-AAM077	R-[(S)-1-(4-bromo-phenyl)-ethylamino]-(2,3-dioxo-1,2,3,4-tetrahydroquinoxalin-5-yl)-methyl]-phosphonic acid
PnTx4-5-5	Γ-ctenitoxin-Pn1a
Ro25-6981	R-(R,S)-α-(4-hydroxyphenyl)-β-methyl-4-(phenylmethyl)-1-piperidine propranol
RP-HPLC	Reverse-phase high-performance liquid chromatography
TCEP	Tris(2-carboxyethyl) phosphine hydrochloride
TUNEL	TdT-mediated dUTP nick-end labeling
UPLC	Ultra-performance liquid chromatography

## References

[1] Monaghan D., Wenthold R. (2012). The Ionotropic Glutamate Receptors.

[2] Furukawa H., Singh S.K., Mancusso R., Gouaux E. (2005). Subunit arrangement and function in NMDA receptors. Nat. Cell Biol..

[3] Yuan H., Hansen K.B., Vance K.M., Ogden K., Traynelis S.F. (2009). Control of NMDA Receptor Function by the NR2 Subunit Amino-Terminal Domain. J. Neurosci..

[4] Hansen K.B., Furukawa H., Traynelis S.F. (2010). Control of assembly and function of glutamate receptors by the amino-terminal domain. Mol. Pharmacol..

[5] Lee C.-H., Lü W., Michel J.C., Goehring A., Du J., Song X., Gouaux E. (2014). NMDA receptor structures reveal subunit arrangement and pore architecture. Nat. Cell Biol..

[6] Song X., Jensen M.Ø., Jogini V., Stein R.A., Lee C.-H., Mchaourab H.S., Shaw D.E., Gouaux E. (2018). Mechanism of NMDA receptor channel block by MK-801 and memantine. Nat. Cell Biol..

[7] Liu L., Wong T.P., Pozza M.F., Lingenhoehl K., Wang Y., Sheng M., Auberson Y.P., Wang Y.T. (2004). Role of NMDA Receptor subtypes in governing the direction of hippocampal synaptic plasticity. Science.

[8] Hardingham G.E., Bading H. (2010). Synaptic versus extrasynaptic NMDA receptor signalling: Implications for neuro-degenerative disorders. Nat. Rev. Neurosci..

[9] Liu Y., Wong T.P., Aarts M., Rooyakkers A., Liu L., Lai T.W., Wu D.C., Lu J., Tymianski M., Craig A.M. (2007). NMDA receptor subunits have differential roles in mediating excitotoxic neuronal death both in vitro and in vivo. J. Neurosci..

[10] Paoletti P., Bellone C., Zhou Q. (2013). NMDA receptor subunit diversity: Impact on receptor properties, synaptic plasticity and disease. Nat. Rev. Neurosci..

[11] Yuan H., Myers S.J., Wells G., Nicholson K.L., Swanger S., Lyuboslavsky P., Tahirovic Y.A., Menaldino D.S., Ganesh T., Wilson L.J. (2015). Context-dependent GluN2B-selective inhibitors of NMDA receptor function are neuroprotective with minimal side effects. Neuron.

[12] Tajima N., Karakas E., Grant T., Simorowski N., Diaz-Avalos R., Grigorieff N., Furukawa H. (2016). Activation of NMDA receptors and the mechanism of inhibition by ifenprodil. Nat. Cell Biol..

[13] Li S.-X., Han Y., Xu L.-Z., Yuan K., Zhang R.-X., Sun C.-Y., Xu D.-F., Yuan M., Deng J.-H., Meng S.-Q. (2018). Uncoupling DAPK1 from NMDA receptor GluN2B subunit exerts rapid antidepressant-like effects. Mol. Psychiatry.

[14] Kovacic P., Somanathan R. (2010). Clinical physiology and mechanism of dizocilpine (MK-801): Electron transfer, radicals, redox metabolites and bioactivity. Oxidative Med. Cell. Longev..

[15] Ito K., Tatebe T., Suzuki K., Hirayama T., Hayakawa M., Kubo H., Tomita T., Makino M. (2017). Memantine reduces the production of amyloid-beta peptides through modulation of amyloid precursor protein trafficking. Eur. J. Pharmacol..

[16] Hackos D.H., Lupardus P.J., Grand T., Chen Y., Wang T.M., Reynen P., Gustafson A., Wallweber H.J., Volgraf M., Sellers B.D. (2016). Positive allosteric modulators of GluN2A-containing NMDARs with distinct modes of action and impacts on circuit function. Neuron.

[17] Volgraf M., Sellers B.D., Jiang Y., Wu G., Ly C.Q., Villemure E., Pastor R.M., Yuen P.W., Lu A., Luo X. (2016). Discovery of GluN2A-selective NMDA receptor positive allosteric modulators (PAMs): Tuning deactivation kinetics via structure-based design. J. Med. Chem..

[18] Karakas E., Simorowski N., Furukawa H. (2011). Subunit arrangement and phenylethanolamine binding in GluN1/GluN2B NMDA receptors. Nat. Cell Biol..

[19] Burger P.B., Yuan H., Karakas E., Geballe M., Furukawa H., Liotta D.C., Snyder J.P., Traynelis S.F. (2012). Mapping the binding of GluN2B-selective N-methyl-D-aspartate receptor negative allosteric modulators. Mol. Pharmacol..

[20] Jones R.M., Bulajm G. (2000). Conotoxins—New Vistas for Peptide Therapeutics. Curr. Pharm. Des..

[21] Estrada G., Villegas E., Corzo G. (2007). Spider venoms: A rich source of acylpolyamines and peptides as new leads for CNS drugs. Nat. Prod. Rep..

[22] Essack M., Bajic V.B., Archer J.A.C. (2012). Conotoxins that confer therapeutic possibilities. Mar. Drugs.

[23] Xiong S., Xu Y., Ma M., Wang H., Wei F., Gu Q., Xu X. (2017). Neuroprotective effects of a novel peptide, FK18, under oxygen-glucose deprivation in SH-SY5Y cells and retinal ischemia in rats via the Akt pathway. Neurochem. Int..

[24] Baig M.H., Ahmad K., Saeed M., Alharbi A.M., Barreto G.E., Ashraf G.M., Choi I. (2018). Peptide based therapeutics and their use for the treatment of neurodegenerative and other diseases. Biomed. Pharmacother..

[25] de Figueiredo S.G., de Lima M.E., Cordeiro M.N., Diniz C.R., Patten D., Halliwell R.F., Gilroy J., Richardson M. (2001). Purification and amino acid sequence of a highly insecticidal toxin from the venom of the Brazilian spider Phoneutria nigriventer which inhibits NMDA-evoked currents in rat hippocampal neurones. Toxicon.

[26] Silva F.R., Batista E.M., Gomez M.V., Kushmerick C., da Silva J.F., Cordeiro M.N., Vieira L.B., Ribeiro F.M. (2016). The *Phoneutria nigriventer* spider toxin, PnTx4-5-5, promotes neuronal survival by blocking NMDA receptors. Toxicon.

[27] Chiu L.S., Anderton R.S., Cross J.L., Clark V.W., Edwards A., Knuckey N.W., Meloni B.P. (2017). Assessment of R18, COG1410, and APP96-110 in excitotoxicity and traumatic brain injury. Transl. Neurosci..

[28] MacDougall G., Anderton R.S., Edwards A., Knuckey N.W., Meloni B.P. (2016). The neuroprotective peptide poly-arginine-12 (R12) reduces cell surface levels of NMDA NR2B receptor subunit in cortical neurons; investigation into the involvement of endocytic mechanisms. J. Mol. Neurosci..

[29] Mir R., Karim S., Kamal M.A., Wilson C.M., Mirza Z. (2016). Conotoxins: Structure, therapeutic potential and pharmacological applications. Curr. Pharm. Des..

[30] Gao B., Peng C., Yang J., Yi Y., Zhang J., Shi Q. (2007). Cone Snails: A Big Store of Conotoxins for Novel Drug Discovery. Toxins.

[31] Prashanth J.R., Dutertre S., Lewis R.J. (2017). Pharmacology of predatory and defensive venom peptides in cone snails. Mol. BioSyst..

[32] Himaya S.W.A., Lewis R.J. (2018). Venomics-accelerated cone snail venom peptide discovery. Int. J. Mol. Sci..

[33] Shen H., Yuan Y., Ding F., Liu J., Gu X. (2008). The protective effects of Achyranthes bidentata polypeptides against NMDA-induced cell apoptosis in cultured hippocampal neurons through differential modulation of NR2A- and NR2B-containing NMDA receptors. Brain Res. Bull..

[34] Shen H., Yuan Y., Ding F., Hu N., Liu J., Gu X. (2010). Achyranthes bidentata polypeptides confer neuroprotection through inhibition of reactive oxygen species production, Bax expression, and mitochondrial dysfunction induced by over-stimulation of N-methyl-D-aspartate receptors. J. Neurosci. Res..

[35] Shen Y., Zhang Q., Gao X., Ding F. (2011). An active fraction of Achyranthes bidentata polypeptides prevents apoptosis induced by serum deprivation in SH-SY5Y cells through activation of PI3K/Akt/Gsk3beta pathways. Neurochem. Res..

[36] Shen H., Wu X., Zhu Y., Sun H. (2013). Intravenous administration of achyranthes bidentata polypeptides supports recovery from experimental ischemic stroke in vivo. PLoS ONE.

[37] Yuan Y., Shen H., Yao J., Hu N., Ding F., Gu X. (2010). The protective effects of Achyranthes bidentata polypeptides in an experimental model of mouse sciatic nerve crush injury. Brain Res. Bull..

[38] Wang Y., Shen W., Yang L., Zhao H., Gu W., Yuan Y. (2013). The protective effects of Achyranthes bidentata polypep-tides on rat sciatic nerve crush injury causes modulation of neurotrophic factors. Neurochem. Res..

[39] Cheng Q., Yuan Y., Sun C., Gu X., Cao Z., Ding F. (2014). Neurotrophic and neuroprotective actions of Achyranthes bidentata polypeptides on cultured dorsal root ganglia of rats and on crushed common peroneal nerve of rabbits. Neurosci. Lett..

[40] Yu S., Wang C., Cheng Q., Xu H., Zhang S., Li L., Zhang Q., Gu X., Ding F. (2014). An active component of Achyranthes bidentata polypeptides provides neuroprotection through inhibition of mitochondrial-dependent apoptotic path-way in cultured neurons and in animal models of cerebral ischemia. PLoS ONE.

[41] Cheng Q., Tong F., Shen Y., He C., Wang C., Ding F. (2019). Achyranthes bidentata polypeptide k improves long-term neurological outcomes through reducing downstream microvascular thrombosis in experimental ischemic stroke. Brain Res..

[42] Peng S., Wang C., Ma J., Jiang K., Jiang Y., Gu X., Sun C. (2018). Achyranthes bidentata polypeptide protects dopaminergic neurons from apoptosis in Parkinson’s disease models both in vitro and in vivo. Br. J. Pharmacol..

[43] Tsai P.L., Chen S.-F., Huang S.Y. (2013). Mass spectrometry-based strategies for protein disulfide bond identification. Rev. Anal. Chem..

[44] Craik D.J., Daly N., Waine C. (2001). The cystine knot motif in toxins and implications for drug design. Toxicon.

[45] Zipfel G.J., Babcock D.J., Lee J.-M., Choi D.W. (2000). Neuronal apoptosis after CNS injury: The roles of glutamate and calcium. J. Neurotrauma.

[46] Kim K.-H., Rodriguez A.M., Carrico P.M., Melendez J.A. (2001). Potential Mechanisms for the inhibition of tumor cell growth by manganese superoxide dismutase. Antioxid. Redox Signal..

[47] Loo D.T. (2010). In situ detection of apoptosis by the TUNEL assay: An overview of techniques. Methods Mol. Biol..

[48] Delivani P., Martin S.J. (2006). Mitochondrial membrane remodeling in apoptosis: An inside story. Cell Death Differ..

[49] Ouyang Y.-B., Giffard R.G. (2004). Cellular neuroprotective mechanisms in cerebral ischemia: Bcl-2 family proteins and protection of mitochondrial function. Cell Calcium.

[50] Kiraz Y., Adan A., Yandim M.K., Baran Y. (2016). Major apoptotic mechanisms and genes involved in apoptosis. Tumor Biol..

[51] Bleich S., Wiltfang J., Kornhuber J. (2003). Glutamate and the glutamate receptor system: A target for drug action. Int. J. Geriatr. Psychiatry.

[52] Lau A., Tymianski M. (2010). Glutamate receptors, neurotoxicity and neurodegeneration. Pflügers Arch. Eur. J. Physiol..

[53] Zhou M., Baudry M. (2006). Developmental changes in NMDA neurotoxicity reflect developmental changes in subunit composition of NMDA receptors. J. Neurosci..

[54] Gascón S., Sobrado M., Roda J.M., Rodríguez-Peña Á., Díaz-Guerra M. (2007). Excitotoxicity and focal cerebral ischemia induce truncation of the NR2A and NR2B subunits of the NMDA receptor and cleavage of the scaffolding protein PSD-95. Mol. Psychiatry.

[55] Ikonomidou C., Turski L. (2002). Why did NMDA receptor antagonists fail clinical trials for stroke and traumatic brain injury?. Lancet Neurol..

[56] Kemp J.A., McKernan R.M. (2002). NMDA receptor pathways as drug targets. Nat. Neurosci..

[57] Mourao C.B., Heghinian M.D., Barbosa E.A., Mari F., Bloch C., Restano-Cassulini R., Possani L.D., Schwartz E.F. (2013). Characterization of a novel peptide toxin from *Acanthoscurria paulensis* spider venom: A distinct cysteine assignment to the HWTX-II family. Biochemistry.

[58] Barbault F., Landon C., Guenneugues M., Meyer J.-P., Schott V., Dimarcq J.-L., Vovelle F. (2003). Solution structure of Alo-3: A new knottin-type antifungal peptide from the insect *Acrocinus longimanus*. Biochemistry.

[59] Heinemann S.H., Leipold E. (2007). Conotoxins of the O-superfamily affecting voltage-gated sodium channels. Cell. Mol. Life Sci..

[60] Traynelis S.F., Wollmuth L.P., McBain C.J., Menniti F., Vance K.M., Ogden K., Hansen K.B., Yuan H., Myers S.J., Dingledine R. (2010). Glutamate receptor ion channels: Structure, regulation, and function. Pharmacol. Rev..

[61] Neyton J., Paoletti P. (2006). Relating NMDA receptor function to receptor subunit composition: Limitations of the pharmacological approach. J. Neurosci..

[62] Zhou Q., Sheng M. (2013). NMDA receptors in nervous system diseases. Neuropharmacol..

[63] Yu X., Khani A., Ye X., Petruzziello F., Gao H., Zhang X., Rainer G. (2015). High-efficiency recognition and identification of disulfide bonded peptides in rat neuropeptidome using targeted electron transfer dissociation tandem mass spectrometry. Anal. Chem..

[64] He Q., Zhang T., Yang Y., Ding F. (2009). In vitro biocompatibility of chitosan-based materials to primary culture of hippocampal neurons. J. Mater. Sci. Mater. Electron..

