# Supplementary material for: Bidentatide, a Novel Plant Peptide Derived from Achyranthes bidentata Blume: Isolation, Characterization, and Neuroprotection through Inhibition of NR2B-Containing NMDA Receptors
Source: Int J Mol Sci. 2021 Jul 26;22(15):7977. doi: 10.3390/ijms22157977 (PMC8348887; doi:10.3390/ijms22157977)
Supplement: Supplementary file 1 [file ijms-22-07977-s001.zip › ijms-1209833-supplementary.pdf]

Supplementary Material

Bidentatide, a Novel Plant Peptide Derived from *Achyranthes bidentata* Blume: Isolation, Characterization, and Neuroprotection through Inhibition of NR2B-Containing NMDA Receptors

Fei Ding [1,†], Yunpeng Bai [2,†], Qiong Cheng [1,†], Shu Yu [1,†], Mengchun Cheng [2], Yulin Wu [2,3], Xiaozhe Zhang [2,4,*], Xinmiao Liang [2,*] and Xiaosong Gu [1,*]

[1] Key Laboratory of Neuroregeneration of Jiangsu and Ministry of Education, Co-Innovation Center of Neuroregeneration, Nantong University, 19 Qixiu Road, Nantong 226001, China; dingfei@ntu.edu.cn (F.D.); cq1981@ntu.edu.cn (Q.C.); yushu@ntu.edu.cn (S.Y.)

[2] CAS Key Laboratory of Separation Sciences of Analytical Chemistry, Dalian Institute of Chemical Physics, Chinese Academy of Sciences, Zhongshan Road 457, Dalian 116023, China; bypnenu@126.com (Y.B.); chengmc@dicp.ac.cn (M.C.); wuyulin0525@163.com (Y.W.)

[3] School of Pharmacy, Henan University of Chinese Medicine, East Jinshui Road 156, Zhengzhou 450046, China

[4] Partner Group of Max Planck Society, Dalian 116023, China

* Correspondence: zhangxz@dicp.ac.cn (X.Z.); liangxm@dicp.ac.cn (X.L.); nervegu@ntu.edu.cn (X.G.)

† These authors contributed equally to this project.

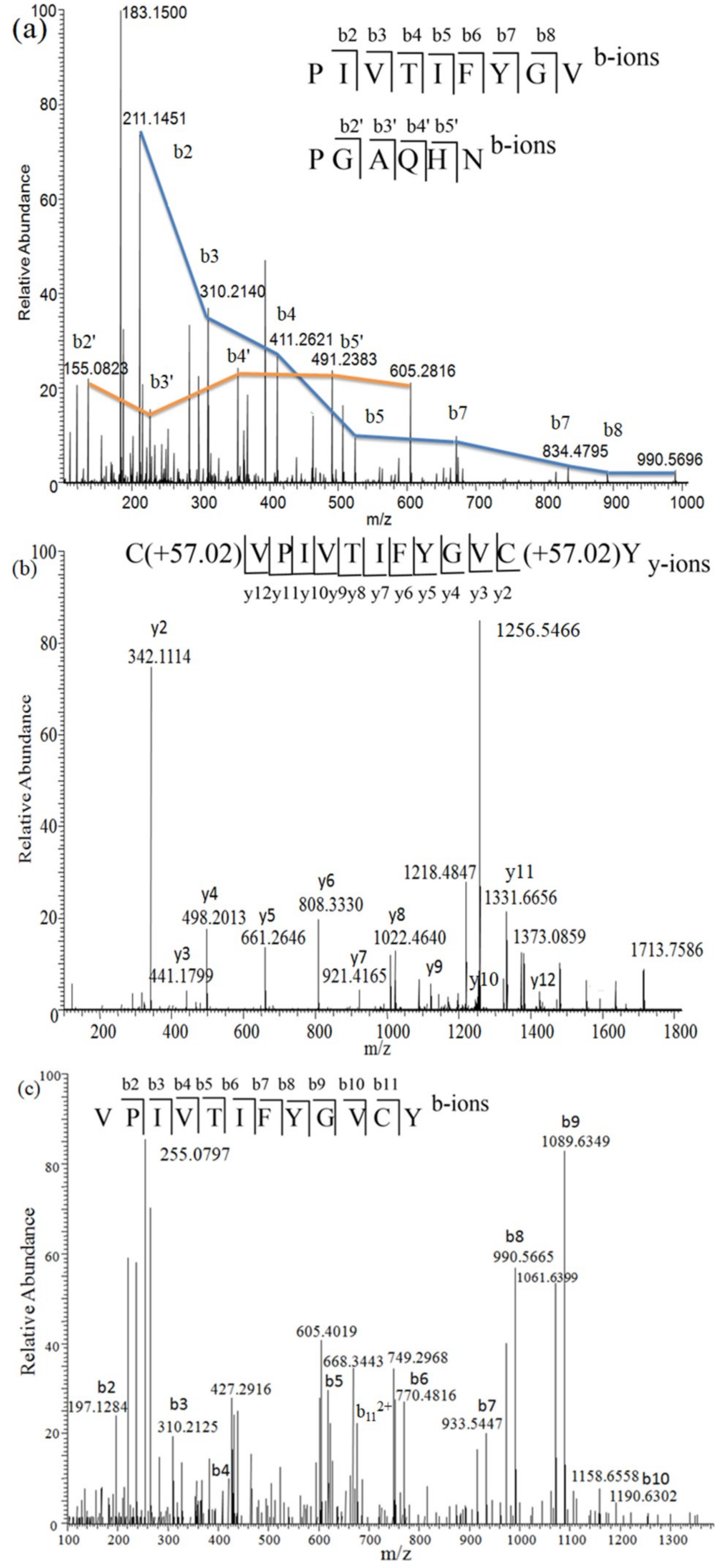
(a)
P I V T I F Y G V b-ions
b2 b3 b4 b5 b6 b7 b8
P G A Q H N b-ions
b2' b3' b4' b5'
183.1500
211.1451
310.2140
411.2621
491.2383
155.0823
605.2816
834.4795
990.5696
Relative Abundance
m/z
(b)
C(+57.02) V P I V T I F Y G V C (+57.02)Y y-ions
y12 y11 y10 y9 y8 y7 y6 y5 y4 y3 y2
342.1114
1256.5466
1218.4847
1331.6656
498.2013
661.2646
808.3330
1022.4640
1373.0859
1713.7586
441.1799
921.4165
Relative Abundance
m/z
(c)
V P I V T I F Y G V C Y b-ions
b2 b3 b4 b5 b6 b7 b8 b9 b10 b11
255.0797
1089.6349
990.5665
1061.6399
605.4019
668.3443
749.2968
770.4816
427.2916
197.1284
310.2125
933.5447
1158.6558
1190.6302
b_{11}^{2+}
Relative Abundance
m/z

Figure S1. MS/MS spectra for bidentatide identification. (a) Internal fragment series from bidentatide, blue line represents PIVTIFYGV, organ line represent PGAQHN; (b) Bidentatide IAM derivative, which represent CVPIVTIFYGVCY ; (c) Acid hydrolysis of bidentatide at 5min, tandem mass spectrometry of peptide segment of VPIVTIFYGVCY-[H2].

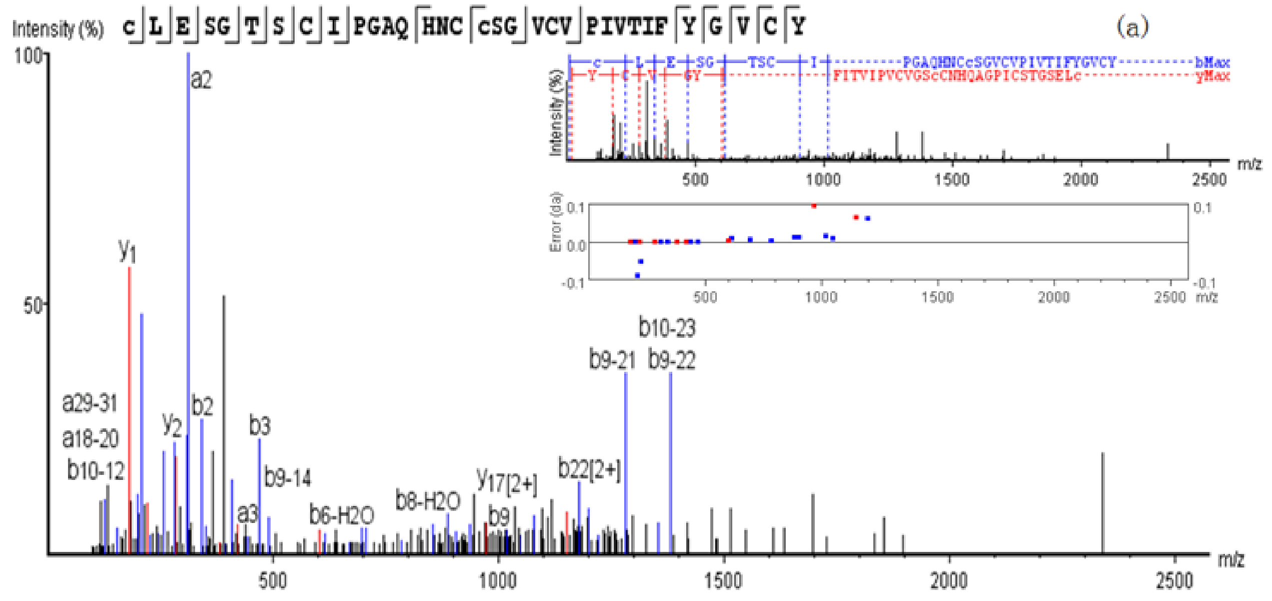

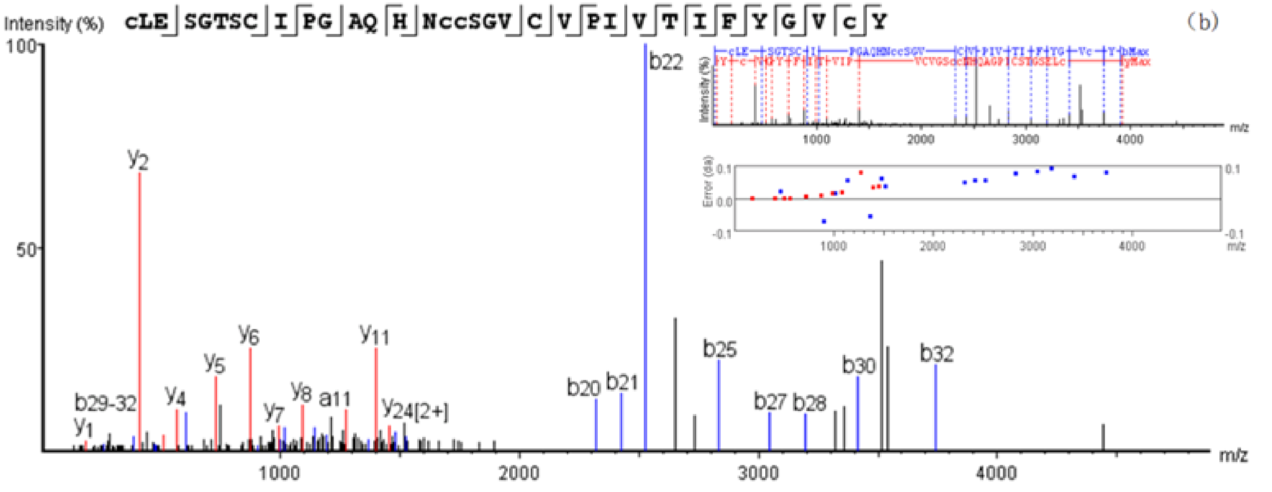

Figure S2. Peaks DB data of bidentatide partial derivative. (a) The disulfide bond between Cys1-Cys17. (b) Disulfide bonds between Cys1-Cys17 and Cys16-Cys32.

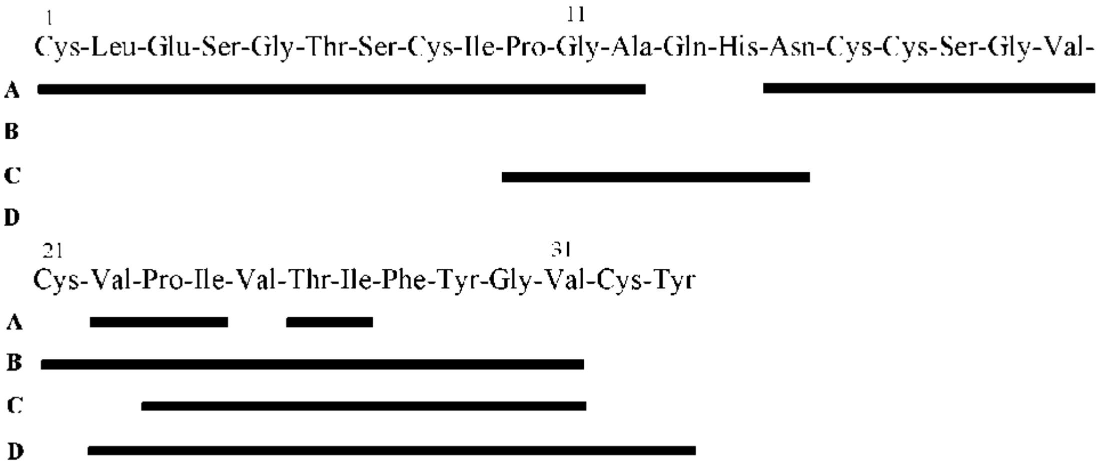

Figure S3. The complete amino acid sequence of bidentatide, with back bars representing identified residues using different methods. (A) Direct sequencing of bidentatide by Edman degradation; (B) MS/MS identification of bidentatide after DTT reduction and IAM alkylation; (C) MS/MS identification of bidentatide by the internal fragment; (D) MS/MS identification of bidentatide after acid hydrolysis.

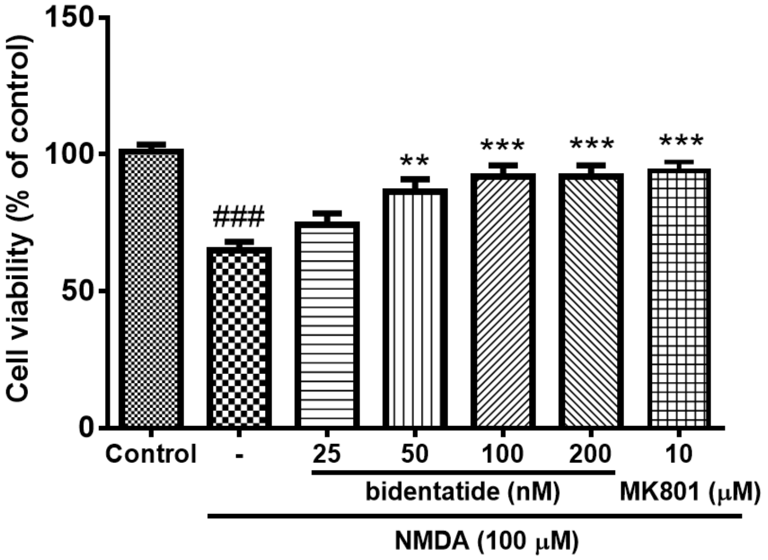

Figure S4. Effects of bidentatide on NMDA-induced cell death in primary cultured hippocampal neurons. Histogram showing the cell viability, as measured by CCK-8 assay, of primary cultured hippocampal neurons upon different cell treatments, including no treatment (control), exposure to 100 μM NMDA stimulation alone, pretreatment with 25-200 nm bidentatide or 10 μM MK801 for 30 min and then exposure to NMDA stimulation. ###p<0.001 versus control; ***p<0.001 and **p<0.01 versus exposure to NMDA alone.

Table S1. Mass spectrum data comparison between bidentatide and its corresponding IAM derivative.

Ion Type	Bidentatide		Bidentatide derivative		Δmass
	m/z(3+)	deconvolution	m/z(3+)	deconvolution	
[M+3H]3+	1139.8247	3416.4522	1255.8803	3764.6190	348.1668
[M+2H+Na]3+	1147.1514	3416.4488	1263.2067	3764.6147	348.1659
[M+2H+K]3+	1152.474		1268.5289		

Table S2. Fragments from precursor ion of Bidentatide. The assignment of amino acid residue compositions in internal fragment series.

Internal fragment series	*m/z*	
Ion Composition	Experimental	[a]Theoretical
PG	155.0823	155.0821
PGA	226.1197	226.1192
PGAQ	354.1789	354.1777
PGAQH	491.2383	491.2367
PGAQHN	605.2816	605.2796
PI	211.1451	211.1447
PIV	310.2140	310.2131
PIVT	411.2621	411.2607
PIVTI	524.3465	524.3448
PIVTIF	671.4155	671.4132
PIVTIFY	834.4795	834.4766
PIVTIFYG	891.5008	891.4980
PIVTIFYGV	990.5696	990.5664

[a] Theoretical *m/z* values of internal fragment series = Σ (amino acid residue) + H^{+}.

Table S3. Fragments from precursor ion of Bidentatide derivative (IAM). The assignment of amino acid residue compositions in y-ion series.

y-ion series	*m/z*	
Ion composition	Experimental	[a]Theoretical
C(+57.02)Y	342.1114	342.1124
VC(+57.02)Y	441.1799	441.1808
GVC(+57.02)Y	498.2022	498.2022
YGVC(+57.02)Y	661.2646	661.2656
FYGVC(+57.02)Y	808.3330	808.3340
IFYGVC(+57.02)Y	921.4165	921.4180
TIFYGVC(+57.02)Y	1022.4640	1022.4657
VTIFYGVC(+57.02)Y	1121.5313	1121.5341
IVTIFYGVC(+57.02)Y	1234.6171	1234.6182
PIVTIFYGVC(+57.02)Y	1331.6656	1331.6710
VPIVTIFYGVC(+57.02)Y	1430.7327	1430.7394
C(+57.02)VPIVTIFYGVC(+57.02)Y	1590.7622	1590.7700

[a] Theoretical *m/z* values of y-ion series = Σ (amino acid residue) + H_3O^{+}.

Table S4. Fragments from peptide segment (RT: 27.78 min) of Bidentatide acid hydrolysis for amino acid sequencing in b-series ion.

Ion composition	Experimental *m/z*	[a]Theoretical*m/z*
VP	197.1284	197.1290
VPI	310.2125	310.2131
VPIV	409.2809	409.2815
VPIVTI	623.4128	623.4132
VPIVTIF	770.4816	770.4816
VPIVTIFY	933.5447	933.5450
VPIVTIFYG	990.5665	990.5664
VPIVTIFYGV	1089.6349	1089.6349
VPIVTIFYGVC-[H_2]	1190.6302	1190.6284
VPIVTIFYGVC-[H_2S]	1158.6558	1158.6563
VPIVTIFYGVCY-[H_2]	686.3555^{2+}	686.3545^{2+}

[a] Theoretical *m/z* values of b-ion series = Σ (amino acid residue) + H^+.
